# Genetic Evidence Reveals Distinct Lineage of Chinese Pangolin in Nepal: Insights From Scat and Blood Samples for Conservation and Wildlife Forensics

**DOI:** 10.1002/ece3.70982

**Published:** 2025-02-16

**Authors:** Fiona Hogan, Faye Wedrowicz, Ambika Pd. Khatiwada, Janardan Dev Joshi, Sam Wasser, Wendy Wright

**Affiliations:** ^1^ Future Regions Research Centre Federation University Australia Victoria Australia; ^2^ Biodiversity Research Institute (University of Oviedo, Principado of Asturias, Spanish National Research Council), Mieres Campus University of Oviedo Mieres Asturias Spain; ^3^ National Trust for Nature Conservation Lalitpur Nepal; ^4^ IUCN SSC Pangolin Specialist Group Zoological Society of London London UK; ^5^ Department of Biology University of Washington Seattle Washington USA

**Keywords:** mitochondrial DNA, non‐invasive genetic sampling, pangolin, scat

## Abstract

Pangolins face critical threats from illegal trade and habitat loss, making their conservation a global priority. Despite their ecological and conservation significance, these elusive creatures remain poorly understood, particularly regarding their phylogeography and genetic diversity. This study successfully isolated DNA from two types of pangolin scat samples (whole scat and swabs) and blood that had been stored frozen for up to 3 years. A mitochondrial *cytochrome b* (424 bp) sequence was reliably generated from both types of scat samples, irrespective of whether the scat appeared in ‘good’ or ‘poor’ condition at the time of collection, and from the blood samples. Sanger sequencing identified four novel *cytochrome b* haplotypes, with distinct variations observed across sampling regions in Nepal (central and east). A comparison with reference sequences from China, Taiwan and Thailand revealed that the Chinese pangolins in Nepal represent a genetically distinct variant, differing by 15–19 base pairs from these other populations. These findings underscore significant genetic differentiation of Chinese pangolins in Nepal, with implications for the species' conservation and management. The methods described in this study are robust and adaptable, offering a valuable framework for broader genetic studies of pangolin populations across Nepal. Such approaches could facilitate the genetic mapping of pangolin variations, aiding in the identification of significant populations, the evaluation of conservation interventions and forensic applications to combat illegal poaching. This work emphasises the critical role of genetics in understanding and protecting pangolins, offering insights that are vital for their long‐term conservation.

## Graphical abstract

In this study, we utilised DNA isolated from stored pangolin scats and blood to amplify the *cytochrome b* region. Comparisons with reference sequences from pangolins originating in China, Taiwan and Thailand revealed a distinct variant of the Chinese pangolin in Nepal. These findings are significant, providing a foundation for future population genetic studies of pangolins in Nepal and informing conservation efforts to combat illegal poaching.

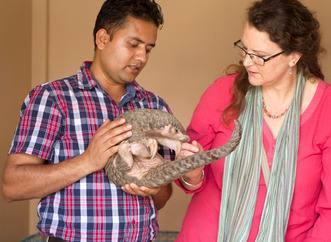

## 1 Introduction

**FIGURE 1:**
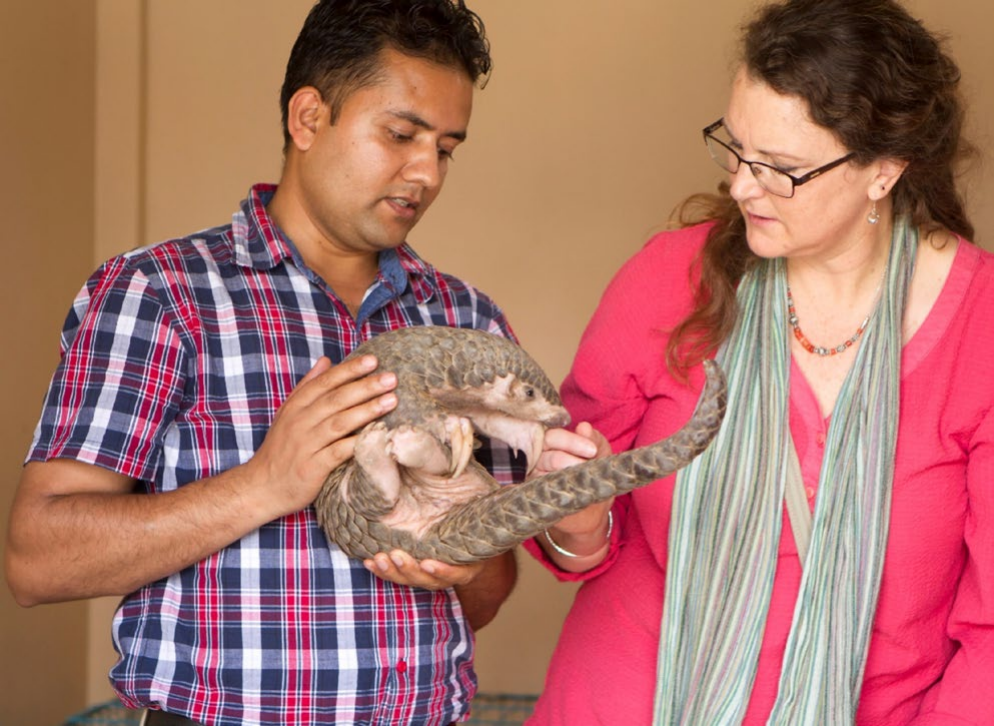
Rescued pangolin seized after a trafficking arrest, housed at the Central Zoo in Kathmandu, held by co‐authors Ambika Khatiwada (left) and Professor Wendy Wright (right). This pangolin was not included in the research presented in this study. Photo credit: Steb Fisher.

The illegal wildlife trade is a major global conservation issue that threatens the future survival of many species, including all eight pangolin species. Pangolins, or scaly anteaters (Order: Pholidota), are typically solitary, nocturnal mammals, found across Asia and Africa. Pangolins have been described as the world's most trafficked mammal group (Aisher 2016), driven by demand for their scales and meat through the illegal wildlife trade (Figure 1). Combating pangolin poaching is challenging due to the ease of capture, the secretive nature of this lucrative trade, associated corruption (Anagnostou and Doberstein 2022) and difficulties in monitoring and protecting pangolins in remote habitats.

**FIGURE 2:**
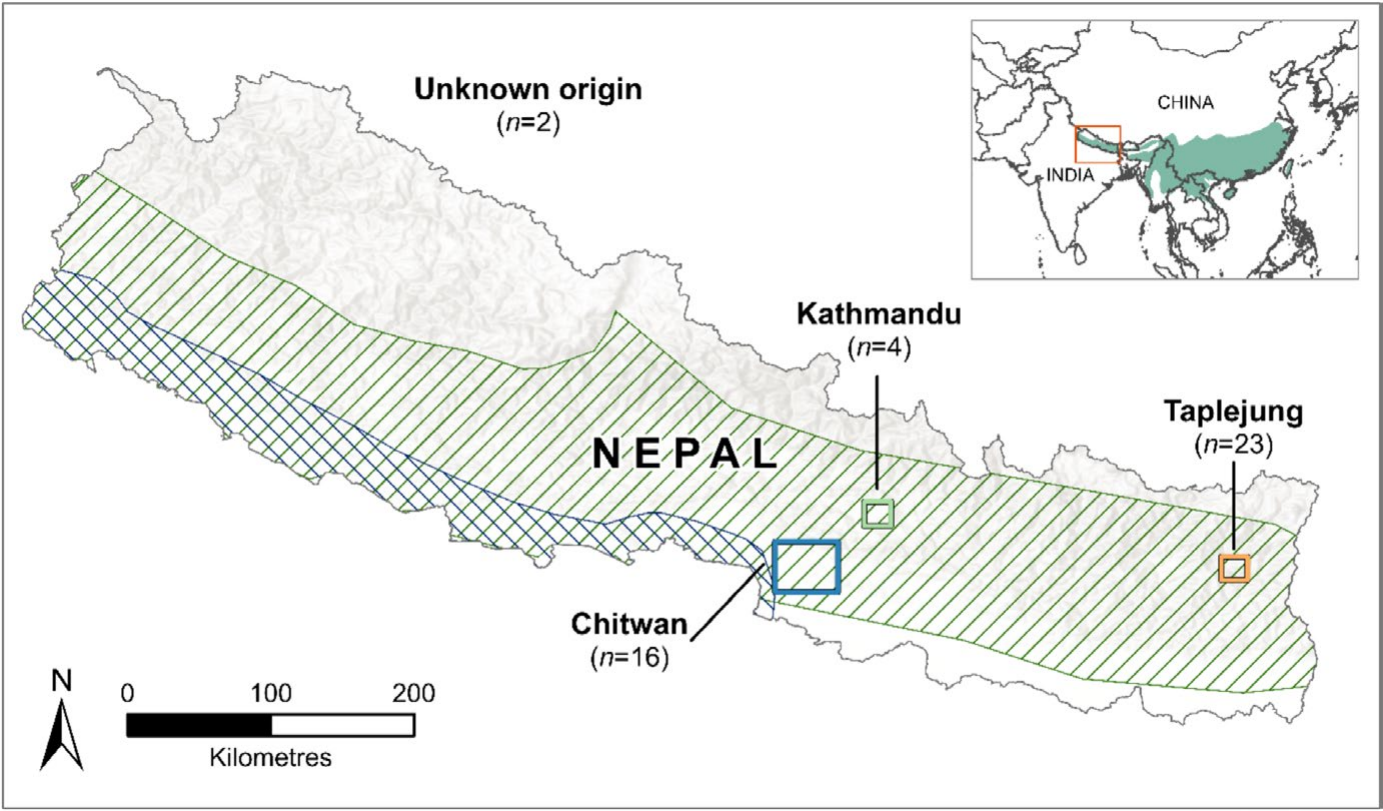
Map of Nepal showing the broad sampling locations for scats (and scat swabs) and the origin of pangolins entering care from which blood samples were taken. Green (forward) diagonal lines indicate the distribution of the Chinese pangolin ( *M. pentadactyla* ) in Nepal, while blue (reverse) diagonal lines show the distribution of the Indian pangolin ( *M. crassicaudata* ) which overlaps with the southern range of the Chinese pangolin in Nepal. The green shading on the inset map shows the full distribution of the Chinese pangolin across Asia.

Four Asian pangolin species are currently recognised: the Sunda Pangolin ( *Manis javanica* ), Philippine Pangolin ( *Manis culionensis* ), Chinese Pangolin ( *Manis pentadactyla* ) and Indian Pangolin ( *Manis crassicaudata* ). Genetic data have recently suggested the existence of a fifth Asian pangolin species (tentatively named *Manis mysteria*); however, these data were derived from seized pangolin material, so the actual geographical origin is unknown (Hu, Roos, et al. 2020; Gu et al. 2023). Two species of pangolin, the Chinese pangolin and the Indian pangolin, occur in Nepal (Khatiwada et al. 2020). Distribution records suggest that Chinese pangolins occur across eastern, central and mid‐western Nepal at elevations of up to 2000 m, while Indian pangolins are found in Nepal's western regions at lower elevations (below 500 m) (Baral and Shah 2008; Jnawali et al. 2011; Sharma et al. 2020; Figure 2). There may be some overlap between the ranges of the two species, though this has not been confirmed (Khatiwada et al. 2020). Nepal, especially eastern Nepal (Ghimire et al. 2020), has long been considered a major hotspot for pangolin poaching and trafficking (Thapa et al. 2014; Katuwal et al. 2015).

Given the threats faced by pangolins, efficient survey and monitoring methods are essential to determine species status, identify priority populations and evaluate conservation interventions. Pangolins are rare, cryptic creatures that are challenging to survey and monitor. While various methods have been explored, including burrow counts, camera trapping and telemetry, molecular techniques are particularly promising for confirming species identification and generating genetic data from scats. Willcox et al. (2019) emphasise the potential of molecular methods but highlight the lack of genetic material from wild pangolins of known geographical origin, which limits the ability to infer the origins of seized pangolin materials through genetic analysis. A reference sample database of georeferenced DNA from wild populations could address this gap, improving the understanding of pangolin population structure and facilitating genetic tracing.

Pietersen and Challender (2020) stress the need for accurate georeferenced DNA databases to identify the geographical origins of trafficked pangolins and guide conservation policy. Molecular techniques have been used in similar contexts for other species to characterise populations across ranges and trace wildlife trade routes (e.g., Wasser et al. 2007; Ghobrial et al. 2010). For pangolins, Gaubert et al. (2016) identified six geographical lineages of the African common pangolin, while Nash et al. (2018) used genetic clusters of Sunda pangolins to infer trade routes. These studies demonstrate the utility of such databases in understanding trafficking dynamics and informing conservation efforts.

Non‐invasive genetic sampling of scats is an effective method for cryptic, threatened species. It allows for broad surveys without capturing or observing the target species, making it cost‐effective, ethical and accessible to non‐experts like natural resource managers or citizen scientists. Scat collection is especially suitable for pangolins, given their elusive nature. Properly preserved scat samples can contribute to long‐term sample banks for genetic research. However, challenges include low target DNA yield, degradation due to environmental factors and contamination. Despite these difficulties, optimised methods for collection and analysis can yield reliable data (Piggott and Taylor 2003; Wedrowicz et al. 2013).

Pangolin scats consist largely of grit and undigested insect matter (Karawita et al. 2020; Mahmood et al. 2021), with target DNA often representing a small proportion of the total DNA. Degradation due to sunlight, moisture and microbial activity further complicates analysis. Nevertheless, advances in techniques for handling low‐quality DNA have demonstrated that genetic data can be effectively extracted from scat samples (Wedrowicz et al. 2019).

This study explores the feasibility of isolating usable DNA from pangolin scats to establish a georeferenced DNA database for wild pangolin populations in Nepal. Using both surface swabs and whole scat material, we aimed to determine whether DNA of sufficient quality and quantity could be obtained. Such a database would help map the geographical extent of pangolin populations and provide a valuable reference for investigations of wildlife crimes such as poaching and trafficking.

## 2 Methods

### 2.1 Sample Origin and DNA Isolation

Between 2017 and 2020, a total of 45 samples (scat and blood) were collected from Chinese pangolins across two broad geographical regions in Nepal: eastern (Taplejung, *n* = 23) and central (Kathmandu, *n* = 4; Chitwan, *n* = 16), as well as two samples from unknown locations, as part of a study conducted by Kim (2021) (Figure 1). Blood samples (*n* = 5) were collected from rescued pangolins, with four from Kathmandu and one from Chitwan. All samples (scat and blood) were stored frozen at −20°C for up to 3 years by Nepal's National Trust for Nature Conservation (NTNC) and provided to us to conduct this study.

Scat swabs were obtained at the time of collection using sterile cotton swabs. Each scat sample's condition was visually assessed during collection and categorised as either ‘good’ or ‘poor’. Samples classified as ‘good’ were fresh and intact with visible moisture, whereas ‘poor’ samples were dry, degraded or mouldy.

Scat samples were categorised into three types: (1) scat material and swab pair from Chitwan (*n* = 11) and Taplejung (*n* = 12); (2) scat material only from Chitwan (*n* = 4), Taplejung (*n* = 8) and unknown locations (*n* = 2); and (3) scat swabs only from Taplejung (*n* = 3) (Table 1).

**TABLE 1:** Summary of sampling locations, sample type and amplification (PCR) and sequencing success using a mitochondrial *cytochrome b* marker.

	Location	Total collected samples	Blood	Paired isolations		Scat material only	Scat swab only	Scat success total
				Scat material	Scat swab			
PCR success	Chitwan	16	100% (1/1)	64% (7/11)	91% (10/11)	0% (0/4)	—	65% (17/26)
	Kathmandu	4	100% (4/4)	—	—	—	—	
	Taplejung	23	—	58% (7/12)	67% (8/12)	75% (6/8)	100% (3/3)	69% (24/35)
	Unknown	2	—	—	—	100% (2/2)	—	100% (2/2)
	**Total**	**45**	**100% (5/5)**	**61% (14/23)**	**78% (18/23)**	**57% (8/14)**	**100% (3/3)**	**68% (43/63)**
Sequencing success	Chitwan	13	100% (1/1)	0% (0/2)	60% (6/10)	—	—	50% (6/12)
	Kathmandu	4	100% (4/4)	—	—	—	—	
	Taplejung	18	—	0% (0/2)	43% (3/7)	83% (5/6)	100% (3/3)	61% (11/18)
	Unknown	2	—	—	—	100% (2/2)	—	100% (2/2)
	**Total**	**37**	**100% (5/5)**	**0% (0/4)**	**53% (9/17)**	**88% (7/8)**	**100% (3/3)**	**59% (19/32)**

*Note:* Total success summaries are highlighted in bold.

DNA was extracted from scat material, scat swabs and blood using standardised protocols with modifications to optimise yield. Frozen whole scat samples were thawed at room temperature. Pieces of scat material were taken from the side of the scat and transferred from each whole scat to a 2‐mL microcentrifuge tube, which was loosely filled to approximately half of the volume of the container (this equated to 200–400 mg of scat material). Scat material was then washed by the addition of 1.5 mL of phosphate‐buffered saline (PBS) to each tube, vortexed thoroughly and placed on a tube rotator for 10 min. The liquid was then transferred to a new tube and centrifuged at 7500 rpm for 5 min. Most of the supernatant was discarded and DNA was then extracted using the QIAamp DNA Stool Mini Kit (Qiagen) with a 1‐h lysis incubation.

Scat swab samples were thawed at room temperature and vortexed for approximately 3 min to release adhered material into the liquid. Tubes were centrifuged at 10,000 rpm for 10 min. A pipette was used to remove most of the supernatant, leaving the pellet undisturbed. Samples were then vortexed and DNA was isolated using the QIAamp DNA Stool Mini Kit (Qiagen). DNA was isolated following the manufacturers protocol except that the sample buffer (ASL) mixture was incubated for 1 h at 35°C to ensure that a homogenous solution was obtained. DNA was eluted using two separate aliquots of 50 μL and combined. DNA was isolated from blood samples using the DNeasy Blood & Tissue Kit (Qiagen) according to the manufacturer's instructions, with DNA eluted in 50 μL.

### 2.2 DNA Quality and Sequencing

A section of the mitochondrial *cytochrome b* (*cytB*) gene was amplified for DNA sequencing using PCR and universal primers L14724 (5′–CGAAGCTTGATATGAAAAACCATCGTTG–3′) and H15149 (5′–AAACTGCAGCCCCTCAGAATGATATTTGTCCTCA–3′) (Kocher and White 1989; Irwin et al. 1991). PCRs were carried out using 1 μM of each primer, 0.1 μg/μL of bovine serum albumin (BSA), 1 × MyTaq Red Mix (Bioline) and 2 μL DNA isolate in a total volume of 20 μL. Thermocycling conditions began with an initial denaturation cycle of 3 mins at 94°C, followed by 40 cycles of denaturation (93°C, 1 min), annealing (50°C, 1 min) and extension (72°C, 1 min) and finished with a final extension cycle of 5 min at 72°C. Amplicons were separated on a 2% agarose gel stained with SYBR safe (Invitrogen) and visualised under UV light.

A subset of samples (*n* = 37) from those that successfully amplified the *cytB* gene were selected for Sanger sequencing blood (*n* = 5), scat material (*n* = 12) and scat swab (*n* = 20) (Table 1). PCR products were purified using ExoSAP‐IT express reagent (Applied Biosystems) following the manufacturer's instructions. DNA sequencing of PCR products was conducted in both directions, and sequences were aligned, trimmed and compared to reference sequences (NCBI BLAST) using Geneious Prime (Geneious 2023.2.1). A minimum spanning haplotype network was produced using the PopART program (Leigh and Bryant 2015) alongside reference haplotypes for *cytB* sequence from the Chinese (MT335859), Indian (MG196306) and Malaysian (MG196309) pangolin (Hari et al. 2016; Gaubert et al. 2018). A phylogenetic tree was also produced to visualise relationships between Chinese pangolin from Nepal and those sampled in other studies/regions. The phylogenetic tree included an additional 14 reference sequences that were retrieved from GenBank to include representatives from all eight species of pangolin and Asian pangolin samples originating from various geographical locations (Table A1). The 17 reference sequences were aligned with the haplotypes identified in this study, along with *cytB* sequence from the gray wolf (HG998573) as an outgroup, trimmed and used to produce a neighbour joining tree with 1000 bootstrap replicates in Geneious Prime.

## 3 Results

### 3.1 Frozen Scat and Swab Samples Provide Sufficient DNA for mtDNA Sequencing

Mitochondrial (*cytB* region) amplification success was reasonable for scat samples with 43/63 (68%) of these samples successfully amplified, and excellent for blood samples: 5/5 (100%), producing clear bands of the expected size (~420 bp, Table 1). Amplification success was greater for DNA isolated from scat swabs (81%, 21/26) than for DNA isolated from scat material taken from whole scats (59%, 22/37; Table 1).

Approximately 70% (28/40) of the scat samples used in this study underwent quality assessments at the time of collection. Among these assessed samples, 22% (9/40) were classified as ‘good’ quality, while the majority, 49% (19/40) were categorised as ‘poor’ quality. These categorisations were based on visual evaluations conducted during sample collection, emphasising the variability in scat condition and its potential influence on downstream DNA analysis.

For scats categorised as ‘good’ quality, at the time of collection, DNA amplification was quite successful, with 100% (6/6) of scat swabs and 63% (5/8) of scat material samples yielding amplified DNA. In contrast, scats classified as ‘poor’ quality showed reduced amplification success, with 78% (11/14) of DNA samples from swabs and 47% (9/19) from scat material resulting in successful amplification. These results (Table 1) highlight the potential impact of scat quality on DNA amplification success.

Sequencing success was highest for DNA isolated from blood, with all five blood samples producing high‐quality sequence data as expected. For scats, success was moderate, with 59% (19/32) of samples yielding readable sequences (Table 1). Quantification of sequence quality was based on two criteria: (1) the number of Q20 bases and (2) signal intensity. For sequences classified as noisy, Q20 scores (reflecting the percentage of bases with a Phred quality score ≥ 20) and signal intensity were typically low, with signal intensity generally below 200. In contrast, readable sequences demonstrated consistently high Q20 scores and signal intensities above 200, indicative of reliable base calling and minimal interference.

Among scats, those collected in Taplejung performed exceptionally well, with 83% of DNA isolated from scat material only and 100% of DNA isolated from scat swabs only producing readable sequence data. DNA isolated from scat swabs, then scat material (pair isolations) from a single scat, did not perform as well as samples where DNA was only isolated from either scat material or scat swab. This result was consistent for samples collected at Chitwan and Taplejung (Table 1).

Scat condition data were available for 26 of the scat samples that were sequenced, with eight classified as ‘good’ and 18 as ‘poor’ condition at the time of collection (Table 2). Sequencing success was higher for scats classified in ‘good’ condition, with 75% (6/8) yielding readable sequence data, compared to just 6% (1/18) for scats in ‘poor’ condition. DNA isolated from swabs showed the highest success rate, with 85% of swabs from scats in good condition producing readable sequences, compared to 7% for swabs from scats in ‘poor’ condition. Conversely, DNA isolated from scat material (*n* = 5) failed to produce readable sequences, though this subset represents a small sample size.

**TABLE 2:** Summary of amplification (PCR) and sequencing success in relation to scat condition for DNA isolated from scat material and scat swabs.

	Scat quality	Scat material	Scat swab
PCR success	Good	63% (5/8)	100% (6/6)
	Poor	47% (9/19)	78% (11/14)
	**All**	**59% (22/37)**	**81% (21/26)**
Sequencing success	Good	0% (0/1)	85% (6/7)
	Poor	0% (0/4)	9% (1/14)
	**All**	**58% (7/12)**	**55% (11/20)**

*Note:* Not all scat samples had associated quality metadata. Total success summaries are highlighted in bold.

### 3.2 Sequence Data Identified Four Unique cytB Haplotypes

**FIGURE 3:**
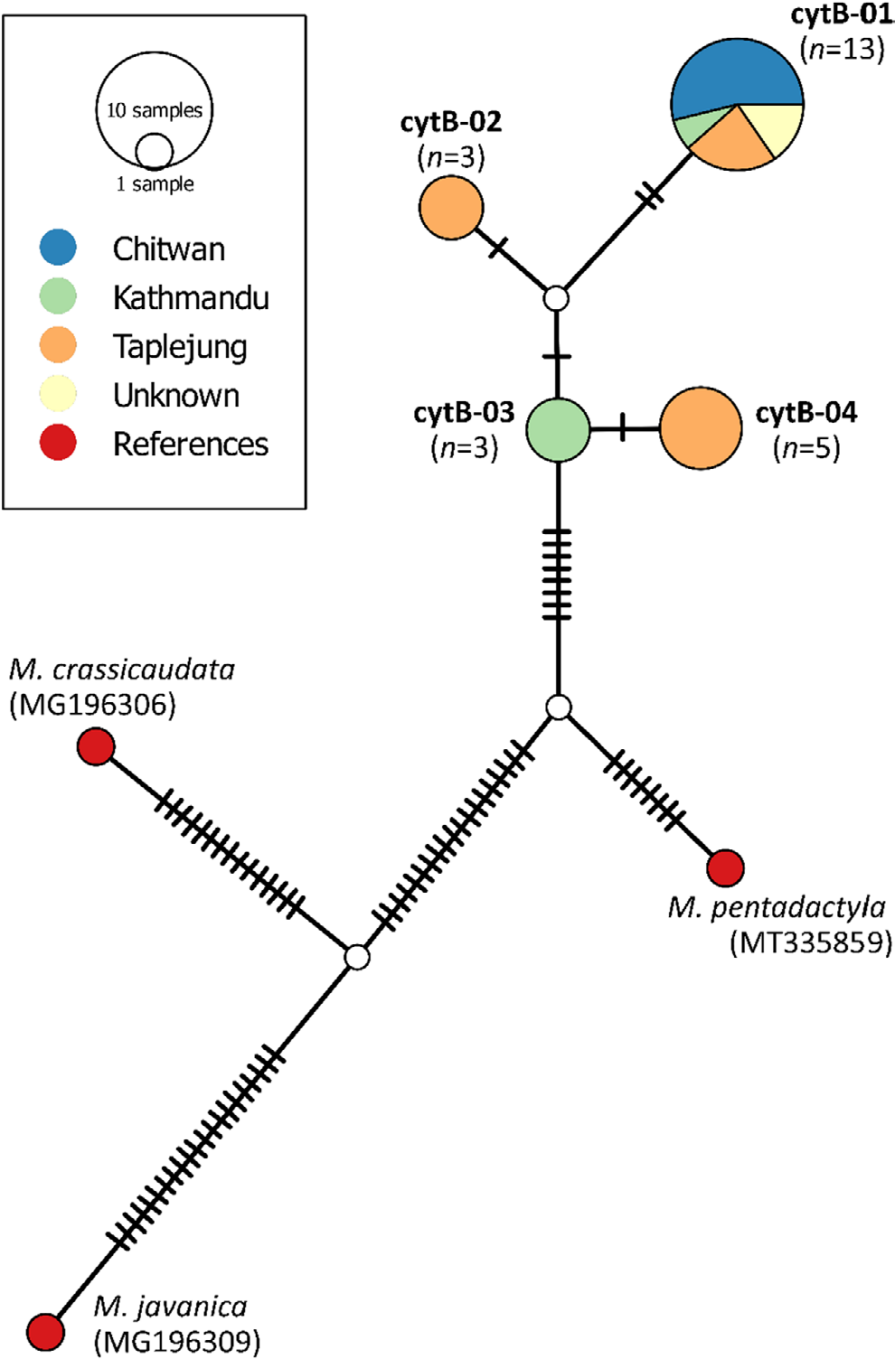
TCS haplotype network showing relationships between the *cytB* haplotypes detected in Nepal (PQ261028, PQ261029, PQ261030, PQ261031) alongside reference haplotypes for the Chinese Pangolin (Guangdong province, China, MT335859), Indian pangolin (Karnataka, India, MG196306) and Malayan pangolin (Guangxi province, China, MG196309).

BLAST comparisons of sequence data indicated that all samples (blood and scat) originated from pangolin and were most similar to the Chinese pangolin. Four new *cytB* haplotypes, cytB‐01 (PQ261028), cytB‐02 (PQ261029), cytB‐03 (PQ261030) and cytB‐04 (PQ261031), were detected within the 24 pangolin *cytB* sequences identified in this study (Figure A3). The most common haplotype detected was cytB‐01, with 54% (13/24) individuals sequenced in this study having this haplotype (Figure 3). Compared to cytB‐01, haplotypes detected in this study had between one and four base pair differences. Other haplotypes were less common and unique to areas in east (Taplejung, cytB‐02 and cytB‐04) or central Nepal (Kathmandu, cytB‐03).

### 3.3 Chinese Pangolins Found in Nepal Have Highly Divergent CytB Sequence

**FIGURE 4:**
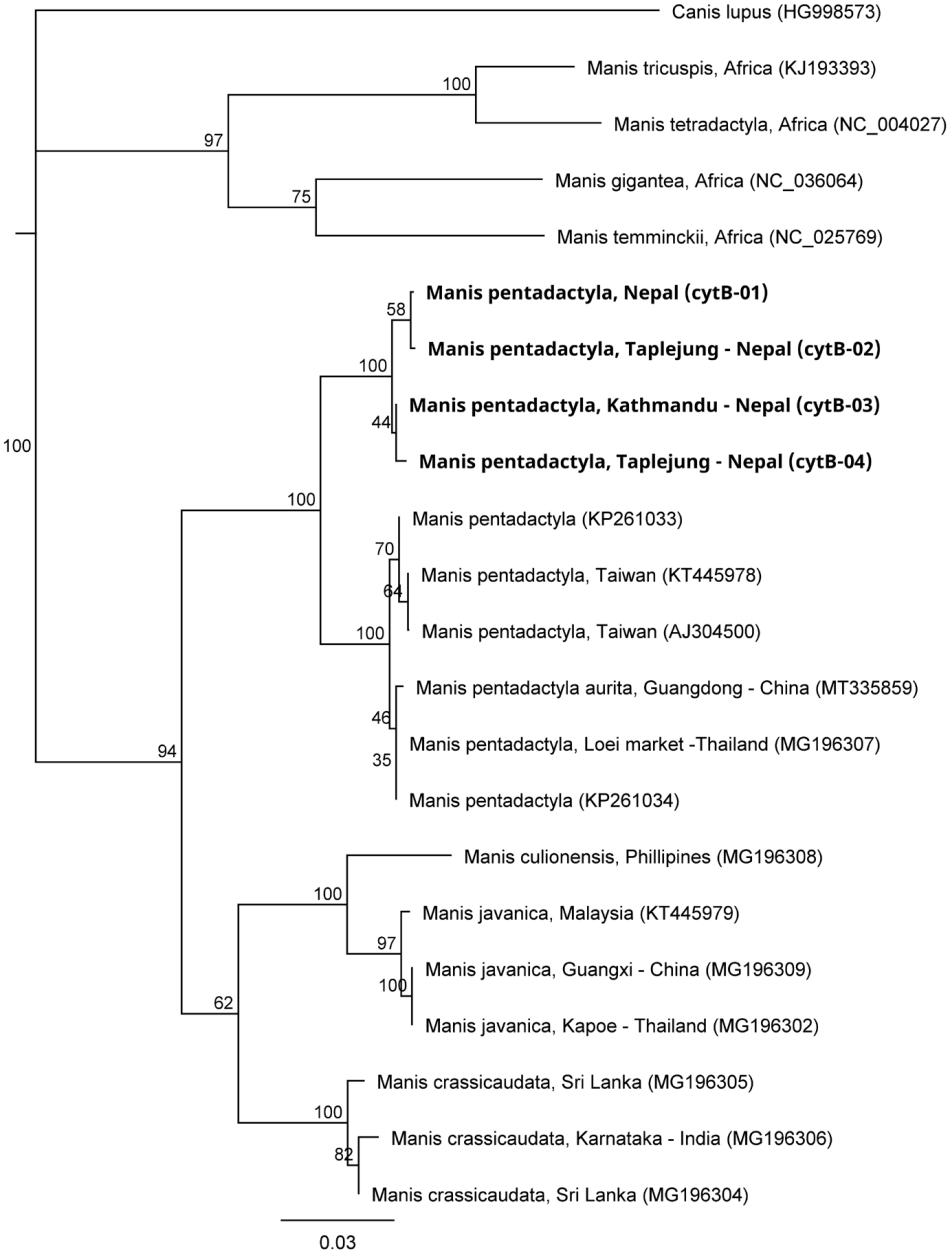
Consensus neighbour‐joining tree based on 1000 bootstraps illustrating the relationships between the partial Chinese pangolin *cytochrome B* haplotypes (424 bp) identified in this study (shown in bold, cytb01 PQ261028, cytb02 PQ261029, cytb03 PQ261030, cytb04 PQ261031) and reference sequences for the eight pangolin species publicly available on Genbank (Table A1). Gray wolf ( *Canis lupus* ) sequence (HG998573) was used as the outgroup.

Comparison of *cytB* sequence obtained from Chinese pangolin originating from Nepal with other publicly available Chinese pangolin sequences showed that Chinese pangolins from Nepal group together in their own subclade (Figure 4). Across 424 bp of *cytB* sequence, the pangolin samples sequenced in this study had between 15 and 19 nucleotide differences (95.5%–96.5% similarity) compared to reference Chinese pangolin sequences sampled in China (Hua et al. 2020), Taiwan (Sun et al. 2021) and Thailand (Gaubert et al. 2018). A neighbour‐joining tree grouped pangolin from Nepal as a sister clade to Chinese pangolin from China (*M. p. aurita*) and Taiwan (*M. p. pentadactyla*) (Figure 4).

**FIGURE A1:**
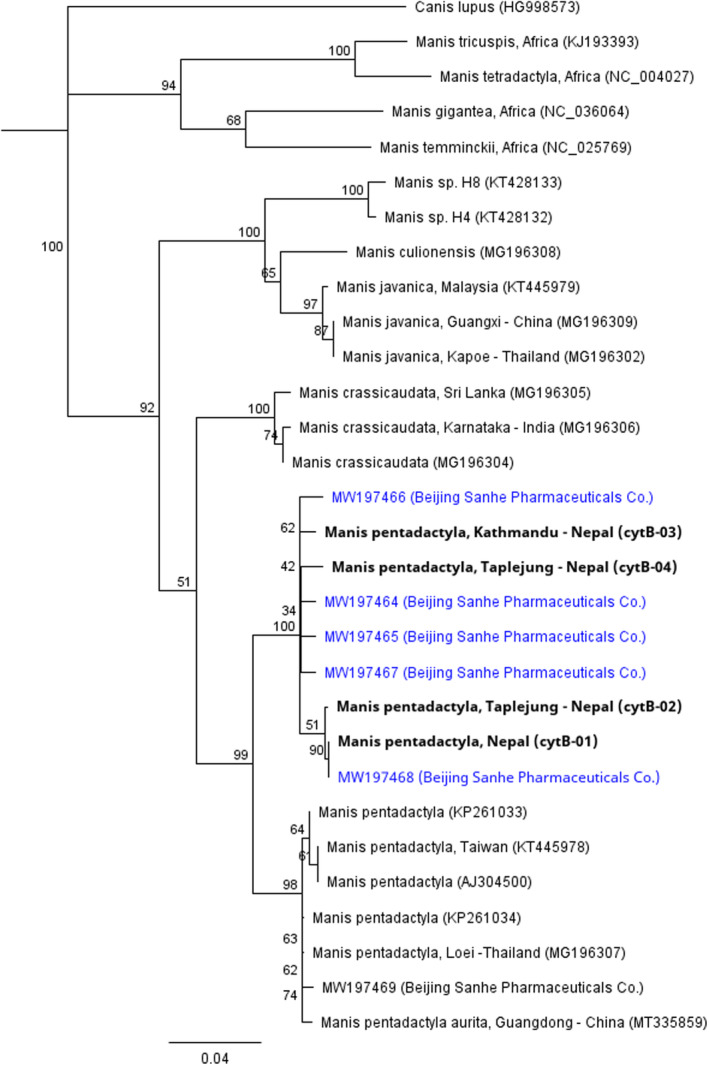
Consensus neighbour joining tree based on 1000 bootstraps illustrating the relationships between the partial Chinese pangolin *cytochrome B* haplotypes (304 bp) identified in this study (shown in bold) in comparison to closest matches identified on GenBank (shown in blue; Xie et al. 2021) and reference sequences. Gray wolf (*Canis lupus*) sequence (HG998573) was used as the outgroup.

**FIGURE A3:**
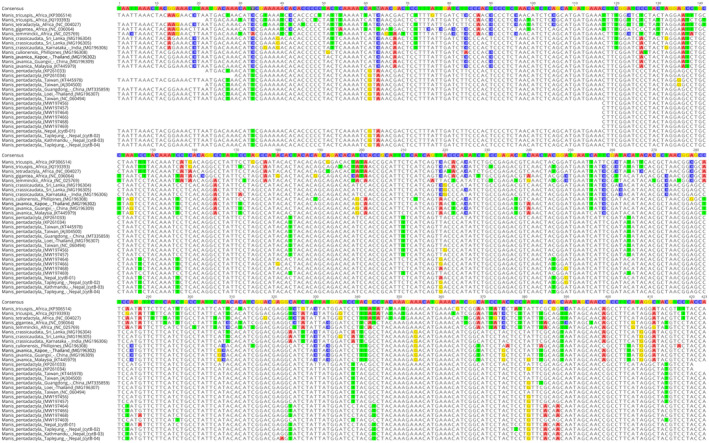
Alignment for the part *cytB* region (423 bp) sequenced in this study along with references obtained from GenBank.

A BLAST search identified five *cytB* sequences on GenBank that were identical or almost identical (1 bp difference) to those in this study (across 304 bp of overlapping sequence, Figure A1). These sequences were reported by Xie et al. (2021) for Chinese pangolin samples that were obtained from a pharmaceutical company located in China (Figure A1).

## 4 Discussion

This study demonstrates that DNA isolated from scat samples can successfully identify the geographical origins of pangolin specimens, highlighting a powerful approach for conservation genetics and wildlife forensics. Our findings show that DNA extracted from scat samples, including those previously frozen, is viable for genetic analysis. Scat samples that appeared fresher at the time of collection provided higher quality DNA, but viable DNA was also obtained from samples in ‘poor’ condition, supporting the utility of including suboptimal samples, particularly from underrepresented regions. The study also suggests that DNA quality may be higher on the scat surface compared to the interior, a consideration that could optimise future sampling protocols.

Furthermore, our findings support the existence of distinct genetic lineages of Chinese pangolins. Specifically, pangolins sampled in Nepal represent a lineage genetically distinct from those in China and Thailand. This conclusion aligns with previous reports (Hu, Hao, et al. 2020; Priyambada et al. 2023) and indicates that at least one unique lineage is present within Nepal. Such differentiation suggests the presence of regionally distinct populations, which may even represent separate subspecies or species, as noted by other studies examining haplotypes and mitochondrial DNA (mtDNA) markers (Shrestha et al. 2020; Priyambada et al. 2023).

### 4.1 Non‐Invasive Sampling and Implications for Conservation

Non‐invasive genetic sampling holds significant value for pangolin conservation, enabling the broad and ethical collection of genetic material without the need to handle or disturb live animals. DNA isolated from scats has proven to be a versatile tool for generating genetic data. For instance, mitochondrial DNA (mtDNA) can provide insights into geographical origins, while nuclear markers, such as microsatellites, offer information on contemporary population substructure (Gossé et al. 2024). Priyambada et al. (2023) demonstrated the utility of this approach by successfully isolating DNA from faecal samples, sequencing a portion of the mtDNA *cytB* gene, and genotyping 20 microsatellite markers. The study reported high amplification success rates (91%–100%) and low genotyping error rates (< 6%), underscoring the reliability of non‐invasive DNA sampling for detailed genetic analysis.

Inferring the geographical origins of seized pangolins or their products with confidence relies on reference data from individuals sampled across their distribution. However, widespread sampling of live, wild pangolins presents significant logistical and ethical challenges. Non‐invasive collection of DNA from scat offers an effective alternative, enabling large‐scale sampling without disrupting natural populations. Establishing a coordinated programme for collecting pangolin scat samples across their range could enable the development of a comprehensive genetic database. Such a resource would be instrumental in identifying critical populations, documenting genetic diversity across landscapes and highlighting populations in need of urgent protection. Additionally, it would provide essential data to inform targeted conservation actions and efforts to combat illegal wildlife trade.

### 4.2 Implications for Forensics

The ability to determine the geographical origins of pangolins is invaluable for identifying poaching hotspots and informing targeted anti‐trafficking efforts. Genetic profiling enables the linkage of seized pangolins or their products to specific locations, significantly enhancing law enforcement strategies and acting as a deterrent to poachers. Moreover, such profiling supports the establishment of a robust forensic framework, where genetic data connect wildlife products to particular crimes and individuals, aiding in successful prosecutions (Wasser et al. 2018, 2022).

Novel molecular techniques, such as those described by Kanokwongnuwut (2018), expand the scope of conservation genetics by enabling the detection of latent DNA, allowing individuals to be associated with specific locations or objects. Chan et al. (2024) illustrated this approach by successfully visualising and amplifying latent DNA deposited by pangolin scales onto plastic packaging materials. Their study demonstrated that DNA isolated from swabs of plastic bags, previously holding pangolin scales could be used to amplify and sequence a portion of the mitochondrial *cytochrome b* locus, showcasing the utility of such methods in forensic and conservation applications.

If a georeferenced genetic database of pangolins across Nepal were developed using methods outlined in this study and by Chan et al. (2024), it could revolutionise forensic science and conservation applications for this endangered species within this region. Such a resource would enable precise identification of pangolins' geographical origins from latent or trace DNA, as well as from sources such as traded bushmeat (Gossé et al. 2024). This capability would allow for accurate tracing of illegal trade routes, offering critical evidence to support conservation law enforcement and aid in prosecution efforts (Ogden, Dawnay, and McEwing 2009). By combining novel molecular techniques with comprehensive genetic mapping, these approaches demonstrate a synergistic potential to bolster pangolin conservation initiatives and significantly enhance anti‐poaching strategies (Heighton et al. 2023).

### 4.3 Challenges and Future Directions

Our results support the use of mitochondrial DNA (mtDNA) for conducting phylogenetic studies of pangolins using DNA isolated from scats. However, as Willcox et al. (2019) suggest, challenges such as the cryptic nature of pangolins, their behaviour of concealing scat traces, and the rapid decomposition of scats in tropical habitats may limit the broader applicability of this method. In this study, the success in locating scats was likely enhanced by using detection dogs (Kim 2021), which facilitated the identification of scat samples more effectively than human‐guided searches. The deployment of trained detection dogs thus mitigates some of the challenges associated with locating scats in the field and highlights their importance as a complementary tool for pangolin conservation research.

Isolating high‐quality DNA from scats also remains challenging due to inhibitors, degradation, and low DNA concentrations. This is evident in this study where approximately 37% of the amplicons from scats failed to provide readable sequence, in contrast to 100% success for quality blood samples. Poor sequencing results observed in this study were likely to be associated with poor amplification or occurred where non‐target DNA was co‐amplified. Further methodological optimisations, such as increasing the amount of scat processed and trialling different DNA isolation kits, could improve DNA yield and quality (Wedrowicz et al. 2019).

Despite the challenges associated with isolating high‐quality DNA from scats, the benefits of using non‐invasive scat sampling in contrast to blood sampling are significant. Scat collection does not require handling or disturbing animals, making it an ethical and less invasive approach, particularly for elusive and vulnerable species like pangolins. This method also allows for broader sampling efforts across larger areas, enabling the collection of genetic material from multiple individuals without the need for direct animal encounters. In contexts where blood sampling may be impractical or raise ethical concerns, scat sampling provides an invaluable alternative. Further optimisation of DNA extraction protocols, and /or redesign of molecular primers to be more specific to pangolins could reduce amplification of non‐target DNA and improve overall sequencing success and data quality. Integration of advanced sequencing technologies, such as next generation sequencing, could also be used to unlock the full potential of scat‐derived DNA for conservation and forensic applications (Andrews et al. 2018).

This study also highlights gaps in our understanding of pangolin genetics. For instance, the genetic divergence observed between populations in Nepal and China exceeds that reported between recognised subspecies of Chinese pangolins, suggesting the need for further investigation into their taxonomy and evolutionary history. Additionally, observed differences in scale morphology and genetic patterns (Zhang and Shi 1991; Wu et al. 2007, Figure A2) may represent unrecognised diversity within Chinese pangolins.

Building a comprehensive DNA database for Chinese pangolins and other pangolin species is a high‐priority conservation effort. Such a resource would enable the characterisation of genetic diversity, identification of critical populations and precise tracing of seized pangolins. This undertaking would require collaboration among governments, research institutions and conservation organisations across Asia.

## Author Contributions

**Fiona Hogan:** conceptualization (equal), writing – original draft (equal), writing – review and editing (equal). **Faye Wedrowicz:** conceptualization (equal), formal analysis (lead), investigation (lead), writing – original draft (equal), writing – review and editing (equal). **Ambika Pd. Khatiwada:** project administration (equal), resources (equal), writing – review and editing (supporting). **Janardan Dev Joshi:** investigation (supporting), writing – review and editing (supporting). **Sam Wasser:** resources (equal), writing – review and editing (supporting). **Wendy Wright:** conceptualization (equal), project administration (equal), writing – original draft (supporting), writing – review and editing (equal).

## Conflicts of Interest

The authors declare no conflicts of interest.

## Figures and Tables

**FIGURE A2:**
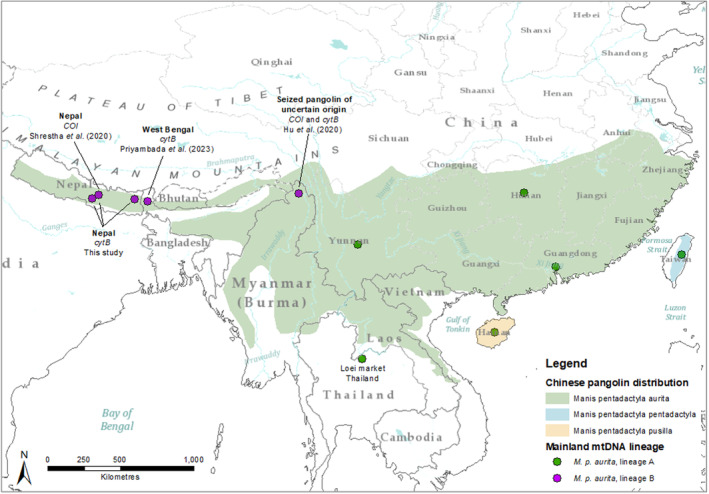
Map illustrating the distribution of the Chinese pangolin and sample sites for studies detecting significantly divergent pangolin lineages. More thorough sampling of Chinese pangolin across their range would be needed to determine whether there are additional lineages and to infer the distributions of the different lineages.

## Data Availability

New *cytB* sequence data are available on GenBank (accession numbers: PQ261028, PQ261029, PQ261030, PQ261031).

## Appendix A

**TABLE A1:** Details of the pangolin reference sequences retrieved from NCBI for use in this study.

Accession	Species name	Common name	Origin	Reference
KP306514	*Manis tricuspis*	White‐bellied pangolin	Africa	Hassanin et al. (2015)
KJ193393	*Manis tricuspis*	White‐bellied pangolin	Africa	Gaubert et al. (2015)
NC_004027	*Manis tetradactyla*	Black‐bellied pangolin	Africa	Arnason et al. (2002)
NC_036064	*Manis gigantea*	Giant ground pangolin	Africa	du Toit et al. (2017)
NC_025769	*Manis temminckii*	Temminck's ground pangolin	Africa	du Toit et al. (2014)
MG196304	*Manis crassicaudata*	Indian pangolin	Sri Lanka	Gaubert et al. (2018)
MG196305	*Manis crassicaudata*	Indian pangolin	Sri Lanka	Gaubert et al. (2018)
MG196306	*Manis crassicaudata*	Indian pangolin	India (Vijayanagara, Ballari, Karnataka)	Gaubert et al. (2018)
MG196308	*Manis culionensis*	Philippine pangolin	Philippines (Iwahig, Palawan Island)	Gaubert et al. (2018)
MG196302	*Manis javanica*	Malayan pangolin	Thailand (Kapoe)	Gaubert et al. (2018)
MG196309	*Manis javanica*	Malayan pangolin	China (Guangxi)	Gaubert et al. (2018)
KT445979	*Manis javanica*	Malayan pangolin	Malaysia	Hari and Choo (unpublished)
KP261033	*Manis pentadactyla*	Chinese pangolin	Southeast Asia	Gaubert and Antunes (2015)
KT445978	*Manis pentadactyla*	Chinese pangolin	Taiwan	Hari et al. (2016)
AJ304500	*Manis pentadactyla*	Chinese pangolin	Taiwan	Lee (unpublished)
MT335859	*Manis pentadactyla*	Chinese pangolin	China (Guangdong)	Hua et al. (2020)
MG196307	*Manis pentadactyla*	Chinese pangolin	Thailand (Loei market)	Gaubert et al. (2018)
KP261034	*Manis pentadactyla*	Chinese pangolin	Southeast Asia	Gaubert and Antunes (2015)
NC_060494	*Manis pentadactyla*	Chinese pangolin	Eastern Taiwan	Sun et al. (2021)
MW197456	*Manis pentadactyla*	Chinese pangolin	Unknown^a^ (1 bp different to MG196307, Thailand)	Xie et al. (2021)
MW197457	*Manis pentadactyla*	Chinese pangolin	Unknown^a^ (equivalent to MG196307, Thailand)	Xie et al. (2021)
MW197464 MW197465 MW197467	*Manis pentadactyla*	Chinese pangolin	Unknown^a^ (equivalent to cytB‐03, Nepal)	Xie et al. (2021)
MW197466	*Manis pentadactyla*	Chinese pangolin	Unknown^a^ (1 bp diff. to cytB‐03, Nepal)	Xie et al. (2021)
MW197468	*Manis pentadactyla*	Chinese pangolin	Unknown^a^ (equivalent to cytB‐01, Nepal)	Xie et al. (2021)
MW197469	*Manis pentadactyla*	Chinese pangolin	Unknown^a^ (1 bp diff. to MG196307, Thailand)	Xie et al. (2021)
PQ261028	*Manis pentadactyla*	Chinese pangolin	Nepal	This study
PQ261029	*Manis pentadactyla*	Chinese pangolin	Nepal	This study
PQ261030	*Manis pentadactyla*	Chinese pangolin	Nepal	This study
PQ261031	*Manis pentadactyla*	Chinese pangolin	Nepal	This study

^a^ Samples of unknown origin sourced from Beijing Sanhe Pharmaceuticals Co. (Xie et al. 2021).
